# Association of lactate-to-albumin ratio with in-hospital and intensive care unit mortality in patients with intracerebral hemorrhage

**DOI:** 10.3389/fneur.2023.1198741

**Published:** 2023-07-13

**Authors:** Dongjie Wu, Siyuan Shen, Dongmei Luo

**Affiliations:** ^1^Anhui University of Technology School of Microelectronics and Data Science, Ma’anshan, Anhui, China; ^2^Anhui Provincial Joint Key Laboratory of Disciplines for Industrial Big Data Analysis and Intelligent Decision, Ma’anshan, Anhui, China

**Keywords:** intracerebral hemorrhage, lactate, albumin, in-hospital mortality, intensive care unit mortality

## Abstract

**Background:**

Intracerebral hemorrhage (ICH) is a severe stroke subtype with a high mortality rate; the lactate-to-albumin ratio (LAR) is a new biomarker for predicting clinical outcomes in patients with ICH. However, the relationship between LAR and mortality in patients with ICH treated in the intensive care unit (ICU) remains controversial. Therefore, in this study, we aimed to investigate the association between LAR and in-hospital and ICU mortality in patients with ICH.

**Methods:**

Patients with ICH were selected from the Medical Information Mart for Intensive Care III (MIMIC-III) database; their clinical information, including baseline characteristics, vital signs, comorbidities, laboratory test results, and scoring systems, was extracted. Univariate and multivariate Cox proportional hazards analyses were used to investigate the association of LAR with in-hospital and ICU mortality. The maximum selection statistical method and subgroup analysis were used to investigate these relationships further. Kaplan–Meier (KM) analysis was used to draw survival curves.

**Results:**

This study enrolled 237 patients with ICH whose lactate and albumin levels, with median values of 1.975 and 3.6 mg/dl, respectively, were measured within the first 24 h after ICU admission. LAR had an association with increased risk of in-hospital mortality [unadjusted hazards ratio (HR), 1.79; 95% confidence interval (CI), 1.32–2.42; *p* < 0.001] and ICU mortality (unadjusted HR, 1.88; 95% CI, 1.38–2.55; *p* < 0.001). A cut-off value of 0.963 mg/dl was used to classify patients into high LAR (≥0.963) and low LAR (<0.963) groups, and survival curves suggested that those two groups had significant survival differences (*p* = 0.0058 and 0.0048, respectively). Furthermore, the high LAR group with ICH had a significantly increased risk of in-hospital and ICU mortality compared to the low LAR group.

**Conclusion:**

Our study suggests that a high LAR is associated with an increased risk of in-hospital and ICU mortality in patients with ICH. Thus, the LAR is a useful prognostic predictor of clinical outcomes in patients with ICH.

## 1 Introduction

Intracerebral hemorrhage (ICH), bleeding caused by a ruptured blood vessel in the brain, is a devastating type of hemorrhagic stroke (1). ICH accounts for 10–20% of all strokes and is associated with more severe morbidity and mortality than ischemic stroke (2, 3). Aggressive blood pressure control, endovascular therapy, and correction of contributing coagulopathies can effectively prevent bleeding (4–6). Although the mortality rate in patients with ICH has decreased significantly in recent decades, it remains stable at approximately 50% (7). Given the high risk of ICH, conducting non-invasive and low-cost tests is necessary to identify patients with a high risk of death and further reduce the mortality rate.

Lactate is produced by red blood cells, transverse muscles, and brain tissues and is excreted by the kidneys. Lactate levels are commonly used to detect tissue hypoxia and reflect the rate of lactate synthesis and metabolism in the liver and kidneys (8). It is a useful biomarker for organ failure (9) and an effective prognostic factor for several critical diseases, including sepsis, trauma, pediatric critical illness, coronavirus disease 2019, and cerebrovascular disease (10–14). Lactate accumulation has been observed in the brains of patients following ICH (15). Studies have explored the prognostic value of lactate in patients with ICH and illustrated that higher lactate levels are associated with 90-day mortality and poor outcomes in patients with ICH (16, 17). Additionally, albumin, the most abundant protein in the plasma, is the main determinant of the balance of plasma oncotic pressure and the main modulator of fluid distribution between body compartments. Patients with ICH experience an acute inflammatory response, leading to decreased serum albumin levels. Administration of serum albumin after ICH improves clinical outcomes (18). A low serum albumin level is associated with increased mortality and poor outcomes in patients with ICH (19, 20). However, lactate and albumin levels can be affected by many factors, such as excessive water in the serum and related comorbidities. Inaccurate levels of lactate and albumin influence the exploration of factors associated with poor outcomes of ICH; therefore, the lactate-to-albumin ratio (LAR) might be a parameter limiting the impact of these factors. Studies have investigated the association of LAR with mortality in diseases, including cardiac arrest, shock, sepsis, and traumatic brain injury (21–23), and LAR was associated with poor outcomes in patients with these diseases. However, the association between LAR and mortality in patients with ICH remains unknown.

**Figure 1:**
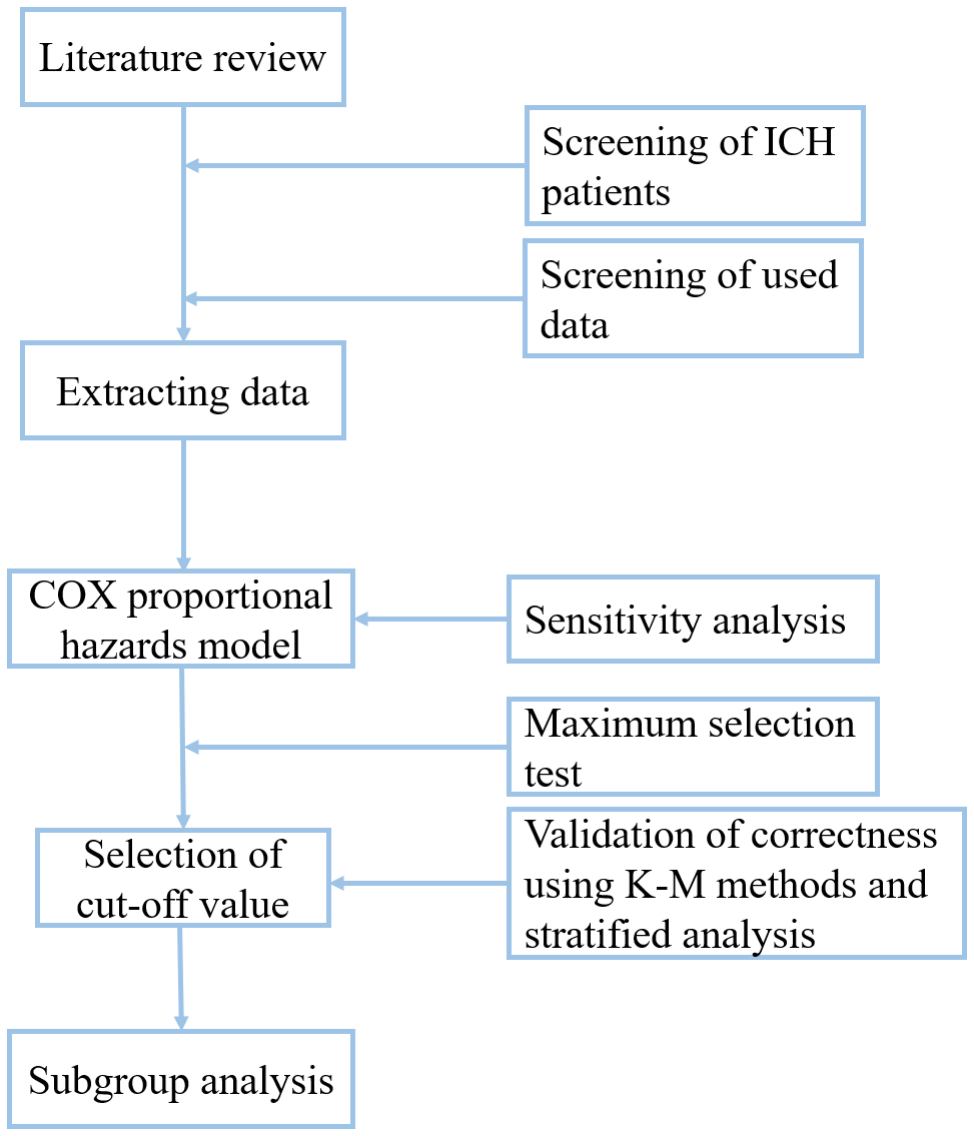
Shows the research flow chart.

This study extracted data on 237 patients with ICH from the Medical Information Mart for Intensive Care III (MIMIC-III) database. The MIMIC-III database contains data on the demographics, vital signs, comorbidities, and laboratory test results of enrolled patients. We aimed to investigate the association of LAR with in-hospital and ICU mortality in patients with ICH. This study could provide a simple and effective indicator for patients with a high risk of ICH by identifying a cut-off value of LAR. The research flow chart was shown in Figure 1.

## 2 Materials and methods

### 2.1 Data source

This retrospective study used the single-center critical care MIMIC-III database (24), de-identified following the Health Insurance Portability and Accountability Act (HIPAA), and it was approved by the Massachusetts Institute of Technology (MIT) and the Beth Israel Deaconess Insurance Center (BIDMC). Each patient provided written informed consent under the principles of the Declaration of Helsinki.

The database contains clinical data, such as demographics, vital signs, laboratory test results, imaging reports, and mortality, associated with 46,520 distinct hospital admissions of adult patients (aged 16 years or older) at the critical care units of the BIDMC between 2001 and 2012 in Boston, Massachusetts. ICU nurses recorded the physiological data obtained from bedside monitors hourly. The present study used the International Classification of Diseases and Ninth Revision (ICD-9) codes for disease diagnosis. The requirement for individual patient consent was waived because the project did not impact clinical care, and all protected health information was de-identified. To have access to the database, users (scientific researchers) have to complete the recognized course: “Protecting Human Research Participants” in the National Institutes of Health’s website. One author, Dong-Jie, completed the course and accessed the database for data extraction.

### 2.2 Population selection

According to the ICD, patients with ICH with an ICD-9 code of 431 were selected for this study. To ensure the accuracy of this study, we excluded patients aged <18 years and those missing important variables, including lactate and albumin data. For patients with multiple hospital and ICU admissions, only those with an ICU admission at the first hospital admission were selected. We used the first recorded value of more than one recorded clinical characteristic in the ICU.

### 2.3 Variable extraction

Structured query language (SQL) with PostgreSQL (version 12.13) was used to extract baseline characteristics, vital signs, comorbidities, laboratory results, and other parameters during the first 24 h of ICU stay. The baseline characteristics included age, sex, weight, ethnicity, insurance, and length of ICU stay. Vital signs included weight, heart rate (HR), respiratory rate (RR), systolic blood pressure (SBP), diastolic blood pressure (DBP), mean blood pressure (MBP), temperature, and percutaneous oxygen saturation (SpO_2_). Information on comorbidities, including congestive heart failure, cardiac arrhythmias, hypertension, diabetes, renal failure, liver disease, and metastatic cancer, was collected for sensitivity analysis based on the ICD-9 code. Laboratory variables were extracted within the first 24 h following ICU admission, including anion gap (AG), bicarbonate, creatinine, chloride, glucose, hematocrit, hemoglobin, platelets, potassium, activated partial thromboplastin time (PTT), international normalized ratio (INR), prothrombin time (PT), sodium, blood urea nitrogen (BUN), and white blood cell (WBC) count. The severity score of each patient was measured using the Oxford acute severity of illness score (OASIS), simplified acute physiology score II (SAPS II), systemic inflammatory response syndrome (SIRS) score, sequential organ failure assessment (SOFA) score, and acute physiology score III (APS III).

### 2.4 Endpoints

Patient follow-up was calculated from the day of hospital admission to the day of death. The clinical endpoints of interest were in-hospital and ICU mortalities. In-hospital mortality was defined as death during hospitalization, and ICU mortality was defined as death during ICU admission.

### 2.5 Statistical analysis

The normality of the data was examined for continuous variables using the Kolmogorov–Smirnov test. Because all continuous variables were not normally distributed, they were expressed as medians (interquartile ranges [IQRs]). Categorical variables were expressed as overall numbers and percentages. Fisher’s test and the chi-square test were used to compare categorical variables between groups (patients with in-hospital death vs. those without in-hospital death; patients with ICU death vs. patients without ICU death). The Wilcoxon rank-sum test was used to compare the continuous variables.

A univariate Cox proportional hazards model was used to investigate the association between LAR and clinical endpoints (risk of in-hospital and ICU mortality). Other potential confounding factors relevant to the clinical endpoints, including age, sex, heart rate, respiratory rate, SBP, DBP, creatinine, AG, WBC, BUN, bicarbonate, glucose, renal failure, and hypertension, were entered into a multivariate Cox proportional hazards model as covariates to further study the association of LAR with these clinical endpoints. As malignancy and insurance status are important causes of death in older individuals, and also considered the potential impact of surgery on outcomes of patients, we also used insurance, metastatic cancer and surgery as covariates in the sensitivity analysis. Thereafter, the maximum selection test method was utilized to determine the cut-off value of LAR for stratified analysis and the association of LAR with the risk of in-hospital mortality and ICU mortality. The Kaplan–Meier (K-M) curve, followed by the log-rank test, was used to visualize relationships. In the stratified analysis, we used two multivariate Cox proportional hazards models adjusted for potential confounders. Model I was adjusted for age, sex, HR, and RR. Model II was adjusted for age, sex, HR, RR, SBP, DBP, AG, creatinine levels, renal failure, and congestive heart failure.

Subgroup analyses of the association of LAR with in-hospital mortality and ICU mortality were further performed using comorbidities (congestive heart failure, cardiac arrhythmias, liver disease, renal failure, and hypertension) and severity scores (SOFA score and SAPS II) in patients with ICH. All statistical analyses were performed using the R software (version 4.1.3). Statistical significance was set at *p* < 0.05.

## 3 Results

### 3.1 Clinical characteristics of the patients at baseline

**Figure 2:**
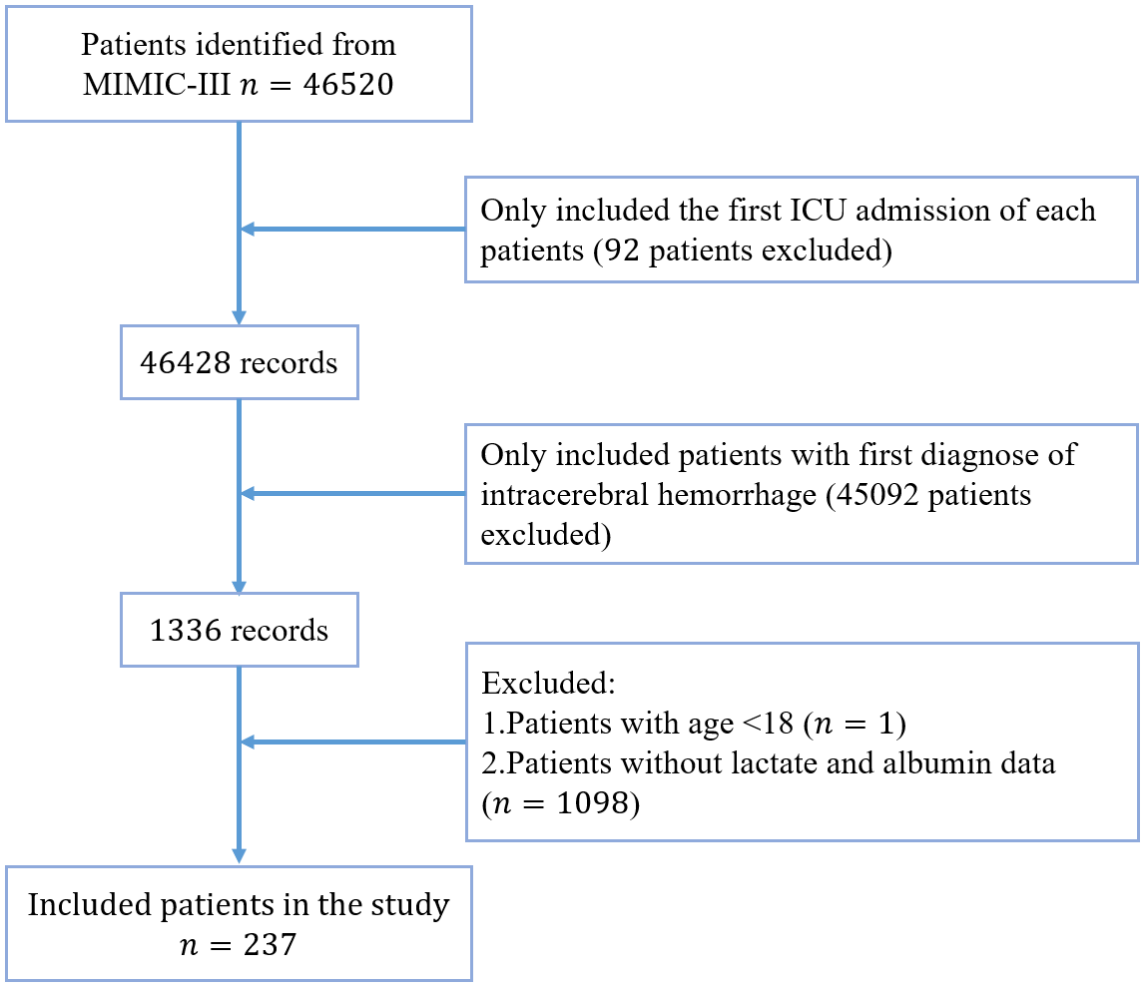
Shows the flow chart of population selection.

Among 46,520 patients in the MIMIC-III database, 237 patients with ICH were selected for this study according to the aforementioned inclusion and exclusion criteria, as shown in the flow chart of population selection (Figure 2). In our study cohort, the median age of the enrolled patients was 64.9 years, and 130 (55%) were male. The in-hospital and ICU mortality rates of the enrolled population were 37.5% (*n* = 89) and 30.8% (*n* = 73), respectively. Among the enrolled patients, 153 (65%) were white, and 20 (8.4%) were black. The median length of ICU stay was 4.2 (2.04–9.78) days. The lactate and albumin test values were measured for all patients in the first 24 h following ICU admission, with median values of 1.98 and 3.60 mg/dl, respectively. The lactate test results between the surviving and non-surviving groups were significantly different: lactate levels in the non-surviving group were significantly higher than those in the surviving group (Supplementary Figure S1). However, albumin levels in the surviving and non-surviving groups were essentially the same, with median values of 3.6 mg/dl. As expected, there was a significant difference in LAR levels between the non-surviving and surviving groups. These data will be useful for our subsequent studies. The baseline characteristics of the enrolled patients, classified according to survival status (alive or dead), are detailed in Table 1. Moreover, patients who died had a longer ICU stay and more comorbidities (diabetes and renal failure) than those who survived. LAR, AG, glucose, WBC, and lactate levels were significantly higher in the individuals who died than in those who survived (*p* < 0.05).

**Table 1:** Baseline characteristics of patients with ICH.

Variable	Overall *N* = 237	In-hospital mortality			ICU mortality		
		Alive *N* = 148	Dead *N* = 89	Value of *p*	Alive *N* = 164	Dead *N* = 73	Value of *p*
Age, years	64.90 (55.81, 76.98)	64.58 (56.51, 76.15)	66.61 (54.44, 78.87)	0.79	65.01 (56.72, 76.83)	64.26 (53.52, 78.08)	0.58
Gender, *n* (%)				0.62			0.77
*F*	107 (45%)	65 (44%)	42 (47%)		73 (45%)	34 (47%)	
*M*	130 (55%)	83 (56%)	47 (53%)		91 (55%)	39 (53%)	
Ethnicity, *n* (%)				0.006			0.016
White	153 (65%)	100 (68%)	53 (60%)		109 (66%)	44 (60%)	
Black	20 (8.4%)	17 (11%)	3 (3.4%)		18 (11%)	2 (2.7%)	
Other	64 (27%)	31 (21%)	33 (37%)		37 (23%)	27 (37%)	
Insurance, *n* (%)				0.59			0.86
Government	8 (3.4%)	7 (4.7%)	1 (1.1%)		7 (4.3%)	1 (1.4%)	
Medicaid	28 (12%)	19 (13%)	9 (10%)		20 (12%)	8 (11%)	
Medicare	120 (51%)	71 (48%)	49 (55%)		83 (51%)	37 (51%)	
Private	75 (32%)	47 (32%)	28 (31%)		50 (30%)	25 (34%)	
Self Pay	6 (2.5%)	4 (2.7%)	2 (2.2%)		4 (2.4%)	2 (2.7%)	
Length of ICU, days	4.20 (2.04, 9.78)	4.85 (2.38, 10.39)	2.76 (1.77, 6.30)	0.006	5.29 (2.62, 11.09)	2.19 (1.63, 5.07)	<0.001
Vital signs							
Weight, kg	76.95 (63.82, 88.00)	77.70 (63.90, 92.60)	75.00 (62.60, 85.00)	0.17	76.20 (63.40, 88.00)	78.10 (66.00, 88.00)	0.80
HR, beats/min	82.83 (72.76, 93.17)	81.12 (71.81, 91.33)	84.54 (75.87, 98.92)	0.029	81.12 (71.89, 91.35)	85.42 (74.73, 99.98)	0.053
SBP, mmHg	132.20 (117.31, 142.19)	133.93 (117.35, 143.50)	127.75 (116.09, 139.92)	0.067	133.01 (117.72, 142.84)	127.51 (109.88, 140.42)	0.10
DBP, mmHg	63.83 (56.25, 71.63)	64.80 (56.55, 73.18)	62.35 (55.06, 69.88)	0.13	65.00 (56.92, 73.00)	62.13 (54.11, 69.88)	0.048
MBP, mmHg	83.17 (76.05, 91.29)	85.36 (77.38, 91.86)	81.79 (74.28, 88.03)	0.015	85.25 (77.61, 91.81)	80.25 (73.44, 87.63)	0.006
Resprate, bpm	18.23 (16.06, 20.61)	18.26 (16.09, 20.56)	18.04 (16.07, 20.62)	0.83	18.18 (15.96, 20.56)	18.33 (16.23, 20.83)	0.50
Temperature, °C	37.06 (36.58, 37.59)	37.03 (36.62, 37.41)	37.19 (36.51, 37.93)	0.12	37.03 (36.59, 37.44)	37.26 (36.51, 37.95)	0.093
SpO_2_, %	98.38 (97.05, 99.31)	98.29 (97.01, 99.29)	98.49 (97.17, 99.36)	0.43	98.39 (97.17, 99.30)	98.38 (96.88, 99.35)	0.66
Comorbidities, *n* (%)							
Congestive heart failure	43 (18%)	29 (20%)	14 (16%)	0.45	32 (20%)	11 (15%)	0.41
Cardiac arrhythmias	68 (29%)	42 (28%)	26 (29%)	0.89	49 (30%)	19 (26%)	0.55
Hypertension	170 (72%)	111 (75%)	59 (66%)	0.15	123 (75%)	47 (64%)	0.094
Diabetes	52 (22%)	29 (20%)	23 (26%)	0.26	30 (18%)	22 (30%)	0.042
Renal failure	23 (9.7%)	11 (7.4%)	12 (13%)	0.13	15 (9.1%)	8 (11%)	0.66
Liver disease	31 (13%)	19 (13%)	12 (13%)	0.89	21 (13%)	10 (14%)	0.85
Metastatic cancer	17 (7.2%)	12 (8.1%)	5 (5.6%)	0.47	13 (7.9%)	4 (5.5%)	0.50
Laboratory results							
Anion Gap, mEq/L	14.67 (13.00, 16.75)	14.50 (12.75, 16.50)	15.50 (13.46, 17.75)	0.021	14.33 (12.75, 16.50)	15.50 (13.50, 18.08)	0.008
Bicarbonate, mEq/L	23.50 (21.15, 26.00)	23.50 (21.33, 26.00)	23.67 (20.92, 25.75)	0.58	23.50 (21.33, 26.00)	23.58 (20.62, 25.54)	0.47
Creatinine, mEq/L	1.00 (0.80, 1.55)	0.96 (0.77, 1.41)	1.13 (0.80, 1.70)	0.062	0.97 (0.80, 1.45)	1.13 (0.78, 1.70)	0.16
Chloride, mEq/L	105.00 (101.67, 108.00)	105.00 (101.88, 107.76)	105.22 (101.67, 108.90)	0.56	105.00 (101.46, 108.00)	105.00 (101.67, 108.67)	0.72
Glucose, mg/dl	143.29 (122.00, 172.17)	141.00 (120.67, 167.08)	152.80 (124.50, 188.33)	0.060	141.00 (119.00, 162.36)	159.40 (126.09, 203.00)	0.005
Hematocrit, %	34.33 (30.80, 38.10)	33.70 (30.62, 37.96)	35.85 (31.78, 38.55)	0.10	33.78 (30.62, 37.93)	35.85 (31.78, 39.12)	0.078
Hemoglobin, g/dl	11.70 (10.43, 13.15)	11.53 (10.37, 12.99)	12.05 (10.70, 13.20)	0.19	11.57 (10.37, 12.96)	12.13 (10.86, 13.30)	0.11
Platelet, K/μl	219.50 (153.33, 277.50)	216.67 (153.83, 275.58)	223.50 (152.00, 283.50)	0.82	218.17 (153.08, 275.12)	223.50 (158.00, 284.00)	0.91
Potassium, mEq/L	3.90 (3.66, 4.20)	3.84 (3.58, 4.21)	3.95 (3.80, 4.20)	0.042	3.87 (3.63, 4.21)	3.93 (3.72, 4.20)	0.39
PTT, s	27.05 (24.70, 31.26)	26.90 (24.73, 29.83)	28.20 (24.00, 35.72)	0.31	27.00 (24.80, 30.10)	27.90 (23.70, 36.47)	0.64
INR	1.20 (1.10, 1.40)	1.20 (1.10, 1.40)	1.20 (1.10, 1.40)	0.78	1.20 (1.10, 1.40)	1.20 (1.10, 1.47)	0.40
PT, s	13.53 (12.70, 15.19)	13.50 (12.70, 15.15)	13.55 (12.80, 15.20)	0.86	13.50 (12.65, 15.10)	13.60 (12.90, 15.70)	0.36
Sodium, mEq/L	139.67 (137.50, 142.00)	139.50 (137.50, 141.38)	140.50 (137.67, 143.20)	0.11	139.50 (137.50, 141.33)	140.67 (137.20, 143.25)	0.081
BUN, mEq/L	18.12 (13.50, 28.33)	17.29 (13.00, 26.42)	19.50 (14.33, 32.80)	0.061	18.00 (13.00, 27.25)	19.00 (14.00, 31.33)	0.40
WBC, K/μl	11.80 (8.73, 15.23)	10.90 (8.16, 14.05)	13.40 (9.73, 16.60)	0.001	10.90 (8.19, 14.04)	13.97 (10.05, 17.15)	<0.001
Lactate, mg/dl	1.98 (1.40, 2.80)	1.90 (1.35, 2.52)	2.20 (1.60, 3.10)	0.010	1.90 (1.40, 2.52)	2.20 (1.50, 3.20)	0.007
Albumin, mg/dl	3.60 (3.10, 4.00)	3.60 (3.14, 3.90)	3.60 (3.10, 4.00)	0.89	3.60 (3.10, 3.90)	3.60 (3.10, 4.00)	0.71
LAR	0.54 (0.41, 0.79)	0.51 (0.39, 0.75)	0.61 (0.44, 0.97)	0.015	0.53 (0.39, 0.76)	0.64 (0.45, 0.98)	0.023
Score system							
OASIS	37.00 (32.00, 43.00)	35.00 (30.75, 40.25)	42.00 (35.00, 47.00)	<0.001	35.00 (31.00, 40.00)	43.00 (36.00, 47.00)	<0.001
SAPS II	38.00 (31.00, 51.00)	36.00 (29.75, 48.00)	46.00 (36.00, 57.00)	<0.001	36.00 (30.00, 48.25)	47.00 (36.00, 58.00)	<0.001
SIRS	3.00 (2.00, 4.00)	3.00 (2.00, 3.00)	3.00 (3.00, 4.00)	<0.001	3.00 (2.00, 3.00)	4.00 (3.00, 4.00)	<0.001
SOFA	4.00 (3.00, 7.00)	4.00 (3.00, 6.00)	5.00 (3.00, 8.00)	0.006	4.00 (3.00, 6.00)	5.00 (4.00, 9.00)	<0.001
APS III	44.00 (30.00, 63.00)	40.50 (28.00, 53.00)	55.00 (33.00, 77.00)	<0.001	41.00 (28.00, 53.25)	60.00 (33.00, 81.00)	<0.001

SBP, systolic blood pressure; DBP, diastolic blood pressure; MBP, mean blood pressure; SpO_2_, percutaneous oxygen saturation; PTT, activated partial thromboplastin time; INR, international normalized ratio; PT, prothrombin time; BUN, blood urea nitrogen; WBC, white blood cell; LAR, lactate-to-albumin ratio; OASIS, oxford acute severity of illness score; SAPS II, simplified acute physiology score II; SIRS, systemic inflammatory response syndrome; SOFA, sequential organ failure assessment; APS III, acute physiology score III.

### 3.2 Association of LAR with in-hospital mortality and ICU mortality

The univariate Cox proportional hazard analysis demonstrated that LAR was significantly poorly associated with increased risk of in-hospital mortality [unadjusted hazard ratio (HR), 1.79; 95% confidence interval (CI), 1.32–2.42; *p* < 0.001] and ICU mortality (unadjusted HR, 1.88; 95% CI, 1.38–2.55; *p* < 0.001) (Table 2). Similar results were obtained after adjusting for age, sex, HR, RR, SBP, DBP, creatinine, AG, WBC, BUN, bicarbonate, glucose, renal failure, and hypertension with covariates in the multivariate Cox proportional hazard model (all HRs > 1 and *p* < 0.05). Based on a sensitivity analysis using insurance coverage, metastatic cancer, and surgery as covariates, we showed that the association of LAR with in-hospital and ICU mortality changed only slightly, suggesting that there was no significant effect of insurance, malignancy, and surgery on the association (Table 3).

**Table 2:** Multivariate Cox proportional hazards analysis for associations between LAR and in-hospital mortality and ICU mortality on patients with ICH.

Variable	In-hospital mortality		ICU mortality	
	HR (95% CI)	Value of *p*	HR (95% CI)	Value of *p*
Crude model				
LAR	1.79 (1.32, 2.42)	<0.001	1.88 (1.38, 2.55)	<0.001
Adjust age				
LAR	1.86 (1.38, 2.51)	<0.001	1.96 (1.44, 2.66)	<0.001
Adjust gender				
LAR	1.86 (1.38, 2.52)	<0.001	1.96 (1.45, 2.66)	<0.001
Adjust heart rate				
LAR	1.76 (1.28, 2.42)	<0.001	1.86 (1.35, 2.57)	<0.001
Adjust respiratory rate				
LAR	1.75 (1.27, 2.41)	<0.001	1.83 (1.32, 2.55)	<0.001
Adjust SBP				
LAR	1.68 (1.19, 2.37)	0.003	1.78 (1.25, 2.53)	0.001
Adjust DBP				
LAR	1.66 (1.17, 2.36)	0.005	1.75 (1.22, 2.51)	0.002
Adjust creatinine				
LAR	1.66 (1.17, 2.36)	0.005	1.76 (1.22, 2.52)	0.002
Adjust anion gap				
LAR	1.59 (1.11, 2.27)	0.011	1.64 (1.13, 2.38)	0.010
Adjust WBC				
LAR	1.59 (1.11, 2.27)	0.011	1.64 (1.13, 2.38)	0.010
Adjust BUN				
LAR	1.64 (1.13, 2.37)	0.009	1.68 (1.15, 2.47)	0.008
Adjust bicarbonate				
LAR	1.70 (1.18, 2.45)	0.005	1.75 (1.19, 2.57)	0.004
Adjust glucose				
LAR	1.66 (1.12, 2.47)	0.013	1.74 (1.15, 2.64)	0.009
Adjust renal failure				
LAR	1.65 (1.10, 2.46)	0.015	1.75 (1.15, 2.65)	0.009
Adjust hypertension				
LAR	1.60 (1.07, 2.40)	0.022	1.68 (1.11, 2.57)	0.015

SBP, systolic blood pressure; DBP, diastolic blood pressure; WBC, white blood cells; BUN, blood urea nitrogen.

**Table 3:** Sensitivity analysis for associations between LAR and in-hospital mortality and ICU mortality on patients with ICH.

Variable	In-hospital mortality		ICU mortality	
	HR (95% CI)	Value of *p*	HR (95% CI)	Value of *p*
Cox model (adjusted insurance)				
LAR	1.65 (1.10, 2.47)	0.015	1.71 (1.12, 2.61)	0.013
Cox model (adjusted malignant)				
LAR	1.65 (1.10, 2.47)	0.016	1.72 (1.12, 2.63)	0.013

Cox model was adjusted for age, gender, HR, RR, SBP, DBP, creatinine, AG, WBC, BUN, bicarbonate, glucose, renal failure and hypertension.

### 3.3 Stratified analysis using a cut-off value for the association of LAR with in-hospital mortality and ICU mortality

To further analyze the association of LAR with in-hospital and ICU mortality, the maximum selection test method was used to determine the cut-off value of LAR. After multiple grouping tests using the chi-square test, the optimal cut-off value corresponding to the maximum statistic was 0.963 (Supplementary Figures S2A,B). Based on the cut-off value, 273 patients with ICH were classified into a low LAR group (<0.963, *n* = 196) and a high LAR group (≥0.963, *n* = 41); demographics, vital signs, comorbidities, laboratory results, and scoring systems of these two groups are shown in Supplementary Table S1. Compared to those in the low LAR group, patients in the high LAR group had the higher risk of in-hospital mortality (56% vs. 34%, *p* = 0.007) and ICU mortality (49% vs. 27%, *p* = 0.006) and significantly high levels of AG, bicarbonate, creatinine, chloride, hematocrit, hemoglobin, platelet, potassium, PTT, INR, PT, sodium, BUN and WBC (*p* < 0.05). Patients in the high LAR group had a higher rate of comorbidities, such as congestive heart failure, diabetes, hypertension, renal failure, liver disease, and metastatic cancer, than patients in the low LAR group.

**Figure 3:**
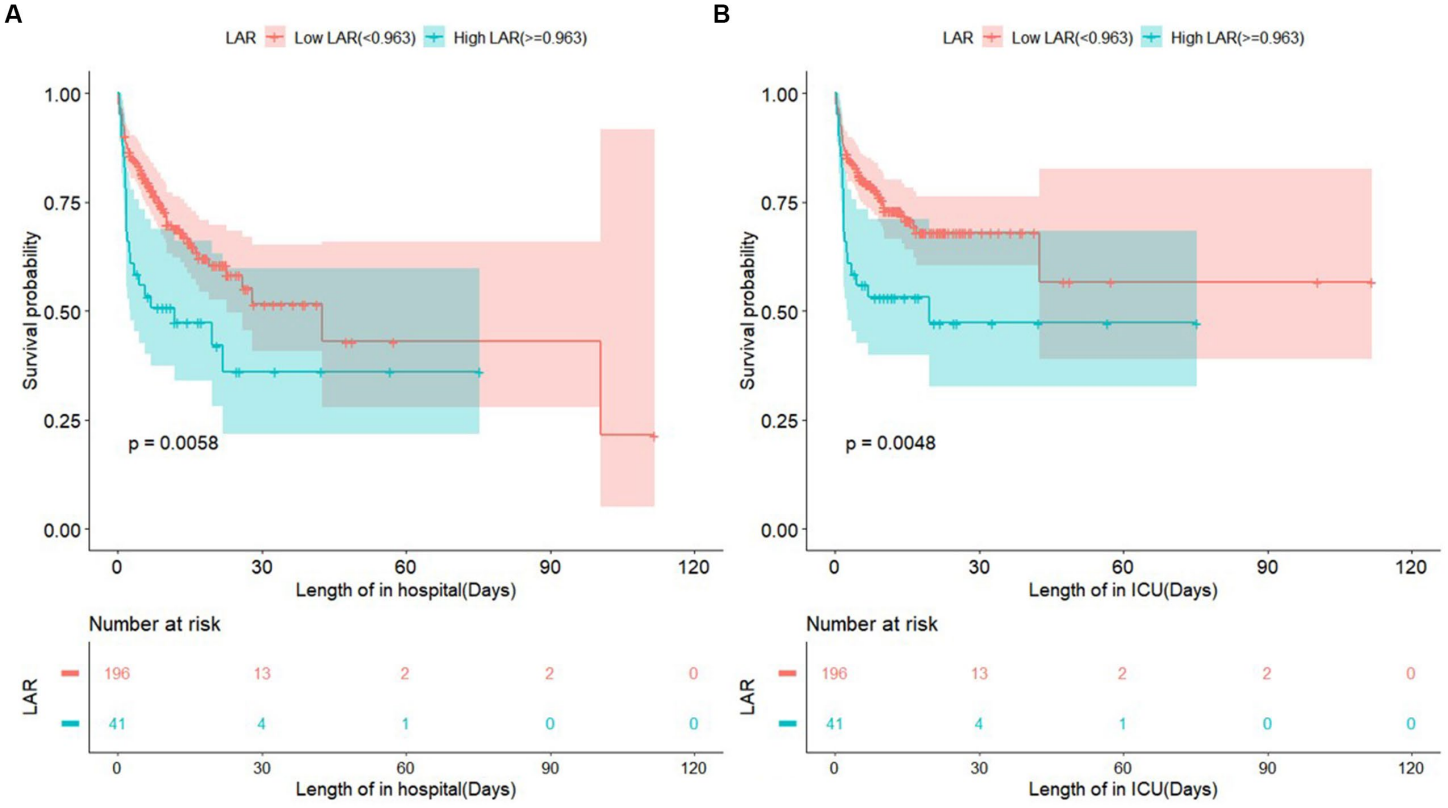
Shows survival curve of in-hospital mortality and ICU mortality classified into two group according to LAR. LAR, lactate to albumin ratio.

To further analyze the association of LAR with in-hospital and ICU mortality, multivariate Cox proportional hazards analyses were used to develop two adjusted models. Model I was adjusted for age, sex, HR, and RR, and Model II was adjusted for age, sex, HR, RR, SBP, DBP, AG, creatinine levels, renal failure, and congestive heart failure. As a continuous variable, LAR was significantly related to in-hospital mortality (Crude model: HR, 1.79; 95% CI, 1.32–2.42; *p* < 0.001; Model I: HR, 1.75; 95% CI, 1.27–2.41; *p* < 0.001; Model II: HR, 1.59; 95% CI, 1.12–2.27; *p* = 0.01) and ICU mortality (Crude model: HR, 1.88; 95% CI, 1.38–2.55; *p* < 0.001; Model I: HR, 1.83; 95% CI, 1.32–2.55; *p* < 0.001; Model II: HR, 1.65; 95% CI, 1.04–2.38; *p* = 0.008). Moreover, as a categorical variable, patients in the high LAR group had a remarkably increased risk of in-hospital mortality (Crude model: HR, 1.93; 95% CI, 1.20–3.11; *p* = 0.007; Model I: HR, 1.90; 95% CI, 1.15–3.12; *p* = 0.012; Model II: HR, 1.77; 95% CI, 1.02–3.06; *p* = 0.041) and ICU mortality (Crude model: HR, 2.07; 95% CI, 1.24–3.46; *p* = 0.006; Model I: HR, 2.06; 95% CI, 1.20–3.53; *p* = 0.009; Model II: HR, 1.88;, 95% CI, 1.04–3.40; *p* = 0.037) compared with those in the low LAR group. It is clear whether LAR as a categorical variable is correlated with a higher risk of in-hospital and ICU mortality than as a continuous variable (Table 4). The K-M curves for patients with in-hospital and ICU mortality are shown in Figures 3A,B; patients in the low LAR group had longer survival times and higher survival rates (log-rank *p* = 0.0048 and 0.0058, respectively) than those in the high LAR group.

**Table 4:** Stratified analysis for association between LAR and in-hospital mortality and ICU mortality in patients with ICH.

Variable	Crude model		Model I		Model II	
	HR (95% CI)	Value of *p*	HR (95% CI)	Value of *p*	HR (95% CI)	Value of *p*
In-hospital mortality						
LAR	1.79 (1.32, 2.42)	<0.001	1.75 (1.27, 2.41)	<0.001	1.59 (1.12, 2.27)	0.010
Low LAR (<0.963)	—		—		—	
High LAR (≥0.963)	1.93 (1.20, 3.11)	0.007	1.90 (1.15, 3.12)	0.012	1.77 (1.02, 3.06)	0.041
ICU mortality						
LAR	1.88 (1.38, 2.55)	<0.001	1.83 (1.32, 2.55)	<0.001	1.65 (1.14, 2.38)	0.008
Low LAR (<0.963)	—		—		—	
High LAR (≥0.963)	2.07 (1.24, 3.46)	0.006	2.06 (1.20, 3.53)	0.009	1.88 (1.04, 3.40)	0.037

ICU, intensive care unit; LAR, Lactate to albumin ratio. Crude model adjusted for none. Model I adjusted for age, gender, heart rate, respiratory rate. Model II adjusted for age, gender, heart rate, respiratory rate, SBP, DBP, anion gap, creatinine, renal failure, congestive heart failure.

### 3.4 Subgroup analysis

A subgroup analysis was conducted to further evaluate the association of LAR with in-hospital and ICU mortality from various perspectives, including comorbidities and severity scores. Our results illustrated that only liver disease significantly interacted with the association of LAR with in-hospital mortality (value of *p* for interaction, 0.013) and ICU mortality (value of *p* for interaction, 0.020). Other comorbidities (congestive heart failure, cardiac arrhythmias, renal failure, hypertension, and diabetes) and severity scores (SOFA score and SAPS II score) had no significant interaction with the association of LAR with in-hospital mortality and ICU mortality (value of *p* for interaction >0.05 in all groups) (Table 5).

**Table 5:** Subgroup analysis by comorbidity and score system for association between LAR and in-hospital mortality and ICU mortality in patients with ICH.

Variables	Overall *N* = 237	In-hospital mortality		ICU mortality	
		LAR ≥0.963 HR (95% CI)	*p* for interaction	LAR ≥0.963 HR (95% CI)	*p* for interaction
Congestive heart failure			0.589		0.272
No	194	1.94 (1.12, 3.33)		2.28 (1.29, 4.04)	
Yes	43	2.92 (0.98, 8.72)		2.13 (0.60, 7.55)	
Cardiac arrhythmias			0.604		0.847
No	169	1.69 (0.95, 3.03)		1.86 (1.01, 3.41)	
Yes	68	2.82 (1.21, 6.57)		3.06 (1.15, 8.20)	
Liver disease			0.013		0.020
No	206	1.35 (0.74, 2.46)		1.41 (0.74, 2.71)	
Yes	31	9.02 (1.94, 41.9)		15.4 (1.95, 123)	
Renal failure			0.451		0.669
No	214	1.88 (1.12, 3.16)		2.00 (1.15, 3.49)	
Yes	23	2.54 (0.74, 8.78)		3.68 (0.81, 16.7)	
Hypertension			0.257		0.486
No	67	2.09 (1.01, 4.33)		1.94 (0.89, 4.24)	
Yes	170	1.62 (0.82, 3.22)		1.94 (0.94, 4.02)	
Diabetes			0.890		0.825
No	185	2.22 (1.27, 3.87)		2.34 (1.27, 4.35)	
Yes	52	1.17 (0.46, 2.98)		1.24 (0.48, 3.20)	
SOFA			0.590		0.985
<4	90	3.42 (1.16, 10.1)		3.13 (0.90, 10.9)	
≥4	147	1.52 (0.88, 2.60)		1.61 (0.91, 2.85)	
SAPS II			0.691		0.916
<38	110	3.61 (1.36, 9.55)		3.81 (1.28, 11.4)	
≥38	127	1.30 (0.75, 2.26)		1.37 (0.76, 2.47)	

SAPS II, simplified acute physiology score II; SOFA, sequential organ failure assessment.

## 4 Discussion

Stroke is a disease that causes disability and death, and worldwide, approximately 10–20% of patients with stroke have ICH (25–27). A study on the assessment of the burden of stroke in China in 2020 found that about 2.1 million patients died of ICH and 0.2 million died of subarachnoid hemorrhage (28). Since there is no effective treatment, early prevention is essential. The present study included more male patients than female patients with ICH, with in-hospital and ICU mortality above 30%. Patients aged 56–75 (median: 65) years were categorized into the high-risk group. Previous studies (29, 30) have also indicated that patients with ICH tend to be younger, and the incidence of ICH is twice as high in males as in females. Several studies have attributed these findings to the higher frequency of alcohol consumption, longer duration of smoking, and greater weight of men than women (29–31). Additionally, hypertension, diabetes, and a previous history of stroke were significant risk factors in ICH patients. Among these risk factors, smoking, alcohol consumption, diabetes, and hypertension can be controlled or treated to reduce the incidence of ICH effectively.

Biomarkers such as blood pressure, cholesterol, and low-density lipoprotein cholesterol and several drugs, such as warfarin, are associated with poor outcomes in patients with ICH. Studies (32–34) that investigated the associations between blood pressure and ICH suggested that hypertension was associated with poor outcomes of patients with ICH and increased risk of death. Aggressive blood pressure control can effectively reduce morbidity and mortality from ICH. Thrift et al. (35) reported that low cholesterol was associated with an increased risk of ICH. Cholesterol may be related to a hemorrhagic tendency that exists throughout the normal range. Hence, we encouraged patients with ICH to reduce their cholesterol intake, which may have implications for ICH mortality risk reduction. Lipids have been suggested to act as biological markers of ICH severity. Satu et al. (36). found that patients with ICH with low-density lipoprotein cholesterol have higher mortality than those with normal levels. Several drugs, such as warfarin, aspirin, and other NSAIDs, have been indicated to be associated with the mortality of patients with ICH (37, 38). The abovementioned biomarkers and drugs can be considered in preventing or treating patients with ICH, contributing to the recovery and reduction of morbidity in patients with ICH.

LAR is a new biomarker of prognostic predictor for patients with various critical illnesses, such as cardiac arrest, sepsis, and trauma (21–23). However, whether the LAR is a prognostic factor in patients with ICH remains uncertain. This study selected 237 patients with ICH from the MIMIC-III database, and their clinical information, including baseline characteristics, vital signs, comorbidities, laboratory test results, and scores, was used to evaluate the severity of ICH. Our study observed poor associations of LAR with in-hospital and ICU mortality rates in patients with ICH. The results of the stratified analysis suggested that the cut-off value of LAR can significantly differentiate between patients with high and low risks; the high LAR group was associated with a significantly increased risk of in-hospital mortality and ICU mortality than the low LAR group.

Furthermore, for patients with ICH with comorbidity of liver disease, the high LAR group was associated with a significantly increased risk of in-hospital mortality and ICU mortality than the low LAR group, possibly due to liver weakening in the patients, leading to changes in the LAR level and, consequently, a higher risk of death. Additionally, our sensitivity analysis showed metastatic cancer, insurance status, and surgery did not significantly affect the independent association of LAR with in-hospital and ICU mortality risks among patients with ICH.

To our knowledge, this study is the first to explore the prognostic value of LAR in patients with ICH. LAR was found to be associated with poor outcomes in patients with ICH admitted to the ICU, and an optimal cut-off value for the LAR was identified. This study provides a new, simple, and convenient biomarker for the identification of ICH that can be used clinically to identify the risk of death in patients with ICH.

The exact mechanism by which LAR is strongly associated with in-hospital mortality in patients with intracerebral hemorrhage is difficult to determine. Several hypothesized mechanisms can be proposed to explain this relationship. First, ICH usually triggers a stress response in the body, which can lead to a disorder in the internal environment. Patients with ICH experience microcirculatory dysfunction and hypermetabolic conditions that lead to lactate accumulation in the brain (39–42). ICH induces an acute inflammatory response due to reduced hepatic synthesis of albumin and increases the endothelial permeability of albumin from the vasculature into the extravascular space, decreasing serum albumin concentration (43–45). Furthermore, unstable brain blood flow leads to changes in lactate and albumin levels. Dehydration is more common in ischemic and hemorrhagic strokes and is related to a high risk of poor outcomes at hospital discharge (46). The onset of dehydration can lead to changes in LAR levels in patients with ICH.

This study has several strengths. First, as we aimed to estimate the real-world prognosis of patients with ICH, we utilized the MIMIC-III database, which contains sufficient clinical information on patients with ICH. Second, we observed that LAR was associated with in-hospital mortality and ICU mortality in patients with ICH, and the high LAR group had a significantly increased mortality risk compared to the low LAR group. Finally, multivariate analysis was performed to control for demographics, laboratory test results, and metastatic cancer, eliminating the influence of relevant confounders. The present study also has a few limitations. First, we only considered the in-hospital mortality and ICU mortality as prognostic indicators; thus, the association of LAR on long-term mortality of patients with ICH should be studied to confirm the prognostic value of LAR further. Second, we excluded 1,062 patients with missing data, and we were uncertain whether this would influence the study results. Finally, we excluded data on drugs and hospital services that may have affected the level of LAR in patients with ICH, which might have influenced our conclusions.

## 5 Conclusion

This study revealed an association between LAR and mortality in patients with ICH. Our results suggest that LAR was a poor prognostic factor for ICH, and a high LAR significantly contributed to an increased risk of in-hospital mortality and ICU mortality than a low LAR in patients with ICH admitted to the ICU. Clinically, LAR can be used as an important biomarker for predicting mortality in patients with ICH.

## Data availability statement

The original contributions presented in the study are included in the article/Supplementary material, further inquiries can be directed to the corresponding author.

## Ethics statement

Ethical review and approval was not required for the study on human participants in accordance with the local legislation and institutional requirements. Written informed consent from the patients/participants or patients/participants’ legal guardian/next of kin was not required to participate in this study in accordance with the national legislation and the institutional requirements.

## Author contributions

SS and DW: conceptualization, data collection, data processing, experiment design, and draft writing. DL: writing – review and editing. All authors have read and agreed to the published version of the manuscript.

## Funding

The present study was funded by the provincial Natural Science Foundation of Anhui (grant no. 1808085MG220), the Research Projects of Anhui Teaching (grant no. 2020jyxm0238); the National Innovation and Entrepreneurship training project (grant no. 202110360094); and the Research Preparation Project of Anhui Province (grant no. 2022AH050328).

## Conflict of interest

The authors declare that the research was conducted in the absence of any commercial or financial relationships that could be construed as a potential conflict of interest.

## Publisher’s note

All claims expressed in this article are solely those of the authors and do not necessarily represent those of their affiliated organizations, or those of the publisher, the editors and the reviewers. Any product that may be evaluated in this article, or claim that may be made by its manufacturer, is not guaranteed or endorsed by the publisher.
