# Supplementary material for: Association of lactate-to-albumin ratio with in-hospital and intensive care unit mortality in patients with intracerebral hemorrhage
Source: Front Neurol. 2023 Jul 13;14:1198741. doi: 10.3389/fneur.2023.1198741 (PMC10374360; doi:10.3389/fneur.2023.1198741)
Supplement: Supplementary file 1 [file Table_1.DOCX]

Table S1 Baseline characteristics of patients with ICH grouped by LAR level

| **Variable** | **Overall**  N = 237 | **Low LAR(<0.963)**  N = 196 | **High LAR(>=0.963)**  N = 41 | **p-value** |
| --- | --- | --- | --- | --- |
| Age, years | 64.90 (55.81, 76.98) | 65.11 (56.09, 78.20) | 64.48 (53.32, 73.50) | 0.33 |
| Gender, n(%) |  |  |  | 0.12 |
| F | 107 (45%) | 93 (47%) | 14 (34%) |  |
| M | 130 (55%) | 103 (53%) | 27 (66%) |  |
| Ethnicity, n(%) |  |  |  | 0.80 |
| White | 153 (65%) | 128 (65%) | 25 (61%) |  |
| Black | 20 (8.4%) | 17 (8.7%) | 3 (7.3%) |  |
| Other | 64 (27%) | 51 (26%) | 13 (32%) |  |
| Insurance, n(%) |  |  |  | 0.96 |
| Government | 8 (3.4%) | 7 (3.6%) | 1 (2.4%) |  |
| Medicaid | 28 (12%) | 23 (12%) | 5 (12%) |  |
| Medicare | 120 (51%) | 101 (52%) | 19 (46%) |  |
| Private | 75 (32%) | 60 (31%) | 15 (37%) |  |
| Self Pay | 6 (2.5%) | 5 (2.6%) | 1 (2.4%) |  |
| Length of ICU, days | 4.20 (2.04, 9.78) | 4.66 (2.10, 9.92) | 2.63 (1.97, 6.11) | 0.084 |
| **Vital signs** | | | | |
| Weight, kg | 76.95 (63.82, 88.00) | 76.20 (63.40, 88.00) | 79.40 (66.10, 89.60) | 0.47 |
| Heart rate, beats/min | 82.83 (72.76, 93.17) | 81.04 (72.21, 91.26) | 91.33 (80.70, 102.95) | <0.001 |
| SBP, mmHg | 132.20 (117.31, 142.19) | 134.39 (120.77, 143.88) | 116.93 (104.12, 132.69) | <0.001 |
| DBP, mmHg | 63.83 (56.25, 71.63) | 65.00 (57.03, 72.89) | 59.62 (52.50, 66.69) | 0.003 |
| MBP, mmHg | 83.17 (76.05, 91.29) | 84.66 (77.46, 91.84) | 78.36 (70.14, 86.75) | <0.001 |
| Respiratory rate, bpm | 18.23 (16.06, 20.61) | 18.06 (16.07, 20.49) | 19.06 (16.05, 21.47) | 0.19 |
| Temperature, ℃ | 37.06 (36.58, 37.59) | 37.06 (36.60, 37.59) | 36.98 (36.53, 37.61) | 0.68 |
| SpO2, % | 98.38 (97.05, 99.31) | 98.47 (97.27, 99.37) | 97.61 (96.18, 99.09) | 0.048 |
| **Comorbidities, n(%)** | | | | |
| Congestive heart failure | 43 (18%) | 32 (16%) | 11 (27%) | 0.11 |
| Cardiac arrhythmias | 68 (29%) | 57 (29%) | 11 (27%) | 0.77 |
| Hypertension | 170 (72%) | 150 (77%) | 20 (49%) | <0.001 |
| Diabetes | 52 (22%) | 40 (20%) | 12 (29%) | 0.21 |
| Renal failure | 23 (9.7%) | 18 (9.2%) | 5 (12%) | 0.56 |
| Liver disease | 31 (13%) | 17 (8.7%) | 14 (34%) | <0.001 |
| Metastatic cancer | 17 (7.2%) | 14 (7.1%) | 3 (7.3%) | >0.99 |
| **Laboratory results** | | | | |
| Anion Gap, mEq/L | 14.67 (13.00, 16.75) | 14.50 (13.00, 16.42) | 16.75 (14.33, 19.33) | 0.002 |
| Bicarbonate, mEq/L | 23.50 (21.15, 26.00) | 24.00 (22.00, 26.00) | 21.00 (16.75, 24.00) | <0.001 |
| Creatinine, mEq/L | 1.00 (0.80, 1.55) | 0.95 (0.75, 1.45) | 1.32 (0.93, 1.75) | 0.002 |
| Chloride, mEq/L | 105.00 (101.67, 108.00) | 104.75 (101.33, 107.76) | 106.50 (103.75, 109.67) | 0.028 |
| Glucose, mg/dL | 143.29 (122.00, 172.17) | 141.10 (122.00, 170.12) | 152.80 (120.33, 180.43) | 0.36 |
| Hematocrit, % | 34.33 (30.80, 38.10) | 34.65 (31.58, 38.58) | 32.50 (28.02, 35.85) | 0.007 |
| Hemoglobin, g/dL | 11.70 (10.43, 13.15) | 11.92 (10.60, 13.22) | 10.70 (9.35, 12.13) | 0.003 |
| Platelet, K/μL | 219.50 (153.33, 277.50) | 224.08 (169.12, 283.62) | 150.80 (83.50, 234.00) | <0.001 |
| Potassium, mEq/L | 3.90 (3.66, 4.20) | 3.86 (3.63, 4.17) | 4.12 (3.77, 4.60) | 0.001 |
| PTT, s | 27.05 (24.70, 31.26) | 26.80 (24.55, 29.50) | 35.78 (25.90, 48.17) | <0.001 |
| INR | 1.20 (1.10, 1.40) | 1.15 (1.10, 1.30) | 1.50 (1.20, 1.92) | <0.001 |
| PT, s | 13.53 (12.70, 15.19) | 13.30 (12.55, 14.64) | 15.53 (14.10, 19.60) | <0.001 |
| Sodium, mEq/L | 139.67 (137.50, 142.00) | 139.67 (137.50, 142.00) | 140.00 (137.50, 142.00) | 0.95 |
| BUN, mEq/L | 18.12 (13.50, 28.33) | 18.00 (13.00, 26.54) | 20.00 (16.00, 33.00) | 0.034 |
| WBC, K/μL | 11.80 (8.73, 15.23) | 11.60 (8.83, 14.72) | 12.90 (7.71, 17.00) | 0.71 |
| LAR | 0.54 (0.41, 0.79) | 0.50 (0.38, 0.67) | 1.38 (1.19, 1.88) | <0.001 |
| **Score system** | | | | |
| OASIS | 37.00 (32.00, 43.00) | 37.00 (31.00, 42.00) | 43.00 (34.00, 45.00) | 0.002 |
| SAPS II | 38.00 (31.00, 51.00) | 37.00 (30.00, 48.00) | 55.00 (44.00, 63.00) | <0.001 |
| SIRS | 3.00 (2.00, 4.00) | 3.00 (2.00, 4.00) | 3.00 (3.00, 4.00) | 0.002 |
| SOFA | 4.00 (3.00, 7.00) | 4.00 (3.00, 6.00) | 8.00 (4.00, 10.00) | <0.001 |
| APS III | 44.00 (30.00, 63.00) | 41.00 (28.00, 56.25) | 68.00 (46.00, 86.00) | <0.001 |
| In-hospital mortality, n(%) | 89 (38%) | 66 (34%) | 23 (56%) | 0.007 |
| ICU mortality, n(%) | 73 (31%) | 53 (27%) | 20 (49%) | 0.006 |
| SBP, systolic blood pressure; DBP, diastolic blood pressure; MBP, mean blood pressure; SpO2, percutaneous oxygen saturation; PTT, activated partial thromboplastin time; INR, international normalized ratio; PT, prothrombin time; BUN, blood urea nitrogen; WBC,white blood cell; LAR, Lactate-to-albumin ratio; OASIS, oxford acute severity of illness score; SAPS II, simplifified acute physiology score II; SIRS, systemic inflammatory response syndrome; SOFA, sequential organ failure assessment; APS III, acute physiology score III. | | | | |
