# Supplementary material for: Association of lactate-to-albumin ratio with in-hospital and intensive care unit mortality in patients with intracerebral hemorrhage
Source: Front Neurol. 2023 Jul 13;14:1198741. doi: 10.3389/fneur.2023.1198741 (PMC10374360; doi:10.3389/fneur.2023.1198741)
Supplement: Supplementary file 3 [file Data_Sheet_2.docx]

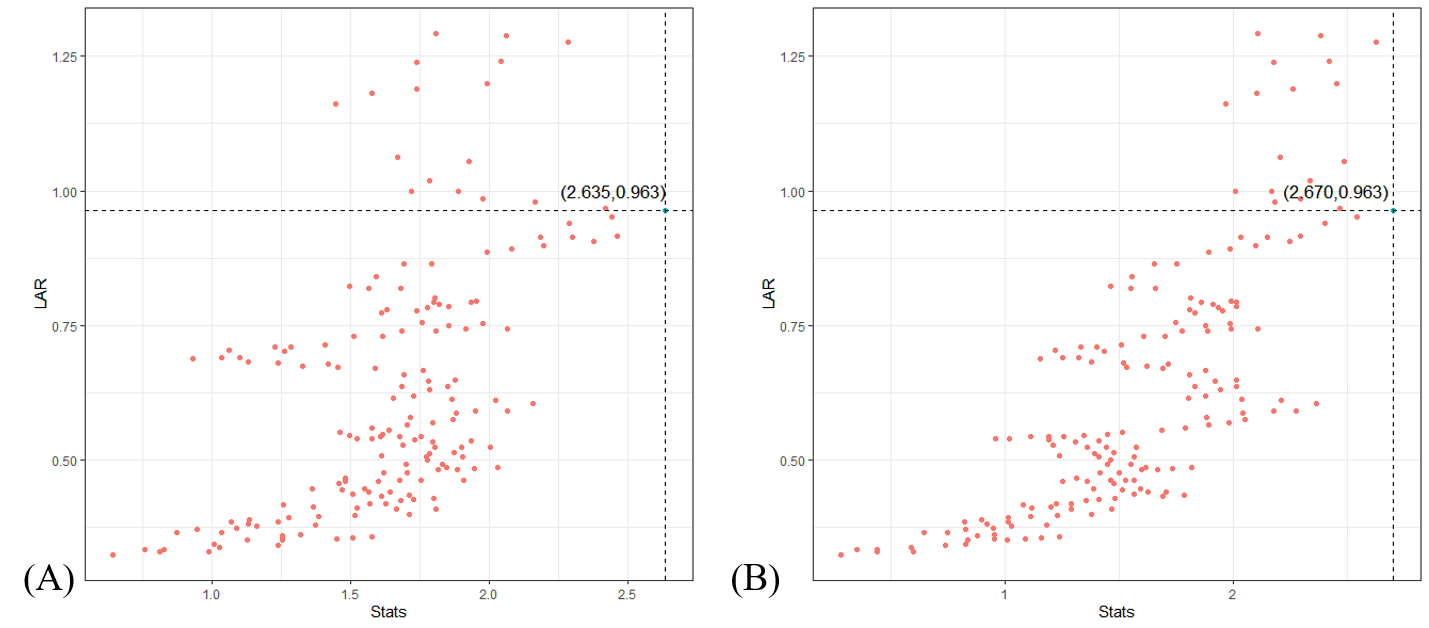


Figure S1 The selection of cut-off value of LAR for in-hospital mortality (A) and ICU mortality (B) in patients with ICH. The figure showed that the cut-off value of LAR is 0.963.


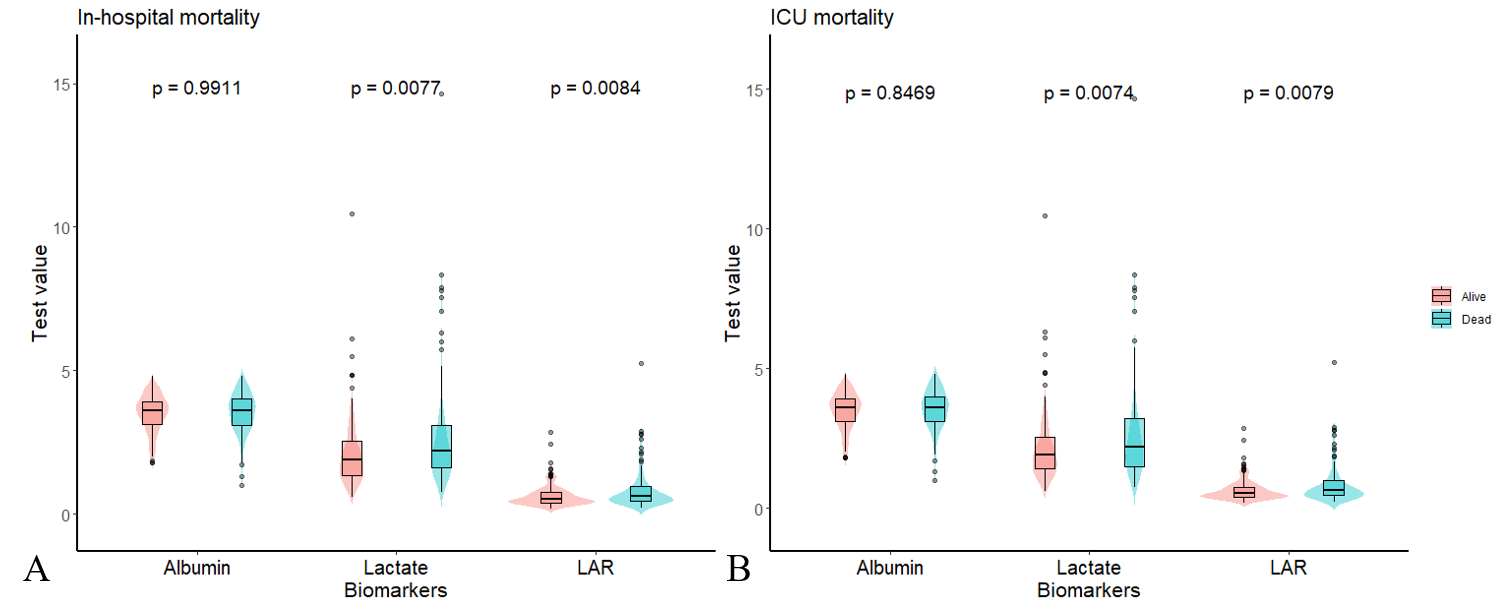


Figure S2 The distribution situation of lactate, albumin and LAR on survival status among in-hospital mortality (A) and ICU mortality (B). There was significant difference between the lactate test values of the surviving and non-surviving groups, and the lactate levels in the non-surviving group being significantly higher than those in the surviving group. However, albumin levels in the surviving and non-surviving groups were essentially the same. Also, there was a significant difference in LAR levels between the non-surviving and surviving groups.
